# Cervical pap smears and pandemics: The effect of COVID-19 on screening uptake & opportunities to improve

**DOI:** 10.1177/17455065211017070

**Published:** 2021-05-25

**Authors:** Hannah Masson

**Affiliations:** School of Medicine, College of Medical, Veterinary and Life Sciences, University of Glasgow, UK

**Keywords:** cervical cancer, cervical screening, colposcopy, COVID-19, HPV, self-sampling

## Abstract

**Background:**

The Coronavirus disease 2019 (COVID-19) pandemic has led to an unprecedented upheaval within global healthcare systems and resulted in the temporary pausing of the National Health Service (NHS) Scotland Cervical Screening Programme. With several months of backlogs in appointments, there has not only been a reduction in primary samples being taken for human papilloma virus (HPV) testing but there have also been fewer women referred to colposcopy for investigation and treatment of precancerous or cancerous changes as a result. Encouraging uptake for cervical screening was always a priority before the pandemic, but it is even more important now, considering that the fears and barriers to screening that women may have are now exacerbated by COVID-19.

**Objectives:**

This article explores the impact of the pandemic on the uptake of cervical screening within NHS Ayrshire & Arran and evaluates potential strategies to improve uptake now and in future such as self-sampling and telemedicine.

**Methods:**

This article presents evidence-based literature and local health board data relating to cervical screening during the pandemic.

**Results:**

Human papilloma virus self-sampling carried out by the woman in her home has been shown to improve uptake, especially in non-attenders, whilst maintaining a high sensitivity and, crucially, reducing the need for face-to-face contact. Increased education is key to overcoming barriers women have to screening and telemedicine can strengthen engagement with women during this time.

**Conclusion:**

There are lessons to be learned from the pandemic, and we must use this opportunity to improve cervical screening uptake for the future.

## Introduction

The COVID-19 (coronavirus) pandemic has brought about the most significant disruption to healthcare services in recent memory, affecting every specialty in medicine. Following the U.K. Government’s announcement of a national lockdown in March 2020 and implementation of social distancing guidance, the priority of the National Health Service (NHS) shifted towards urgent and essential medical care and many clinics, elective surgeries and national screening programmes were temporarily ‘paused’ while the NHS responded to COVID-19. One screening programme affected was the Scottish cervical screening programme which was paused on 30 March 2020 and only resumed taking more urgent appointment bookings from 29 June and routine appointments from September. This essay will explore the challenges and barriers to uptake of cervical screening that the pandemic presented including the effect of the inevitable backlog in diagnosis of cervical cancer, as well as possible future methods of carrying out cervical screening that would reduce the need for face-to-face contact, which is becoming an emerging theme in healthcare and may continue long after the pandemic. The sources used were selected due to their applicability to the Scottish cervical screening programme where possible. Other sources used include guidelines from the U.K. government, Public Health and societies such as the Royal College of Obstetricians & Gynaecologists as well as several peer-reviewed journal articles.

## Cervical screening programme

**Figure 1.**
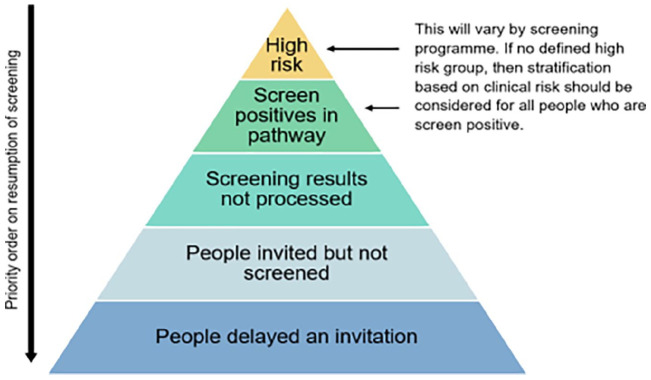
Hierarchy of restart pyramid. Source: From Public Health England.^2^

Cervical screening in Scotland began in the 1960s and is now offered to women aged 25–49 every 3 years and to those aged 50–64 every 5 years.^1^ The main phases of the cervical screening programme are: primary screening of women in the general population, triaged testing of the proportion of women thought to have precancerous or cancerous changes and treatment of these women who have been confirmed by testing as having a greater risk. At present, the order in which appointments are being restarted will give priority to those who had an abnormal result before screening was halted followed by those who did not receive an invitation to screening when they otherwise would have during lockdown. Although this is a logical order in which to triage those most in need, it does mean that women who were due to attend on a routine basis had to wait longer to receive their delayed invitation. The principles of restoration of the cervical screening programme were published by Public Health England and NHS England and NHS Improvement (NHSEI) in April,^2^ and the following Figure 1 shows the prioritization, when services resume, of those at highest risk.

Nearly every case of cervical cancer is caused by infection with an oncogenic strain of human papilloma virus (HPV) with two types (HPV 16 and 18) causing 70% of cancerous and precancerous change in the cervix.^3^ Young girls and more recently boys are offered an HPV vaccine to decrease the transmission of HPV and therefore incidence of cervical cancers. Since March 2020, Scotland has implemented HPV testing to replace cervical cytology as a primary cervical screening test.^1^ This is an evidence-based change as HPV testing has demonstrated higher sensitivity for high-grade cervical intraepithelial neoplasia (CIN) and provides a stronger negative predictive value than cytology, meaning that there is a possibility of safely extending the screening interval following a negative result.^4^ This would be beneficial in future to reduce the strain on screening services currently dealing with a backlog of appointments. Another useful opportunity that primary HPV testing presents is the possibility of introducing self-sampling kits which may help women who find attending for screening difficult, and research is being carried out into the effectiveness of this strategy.

## Cervical screening uptake before and during pandemic

Public Health Scotland published annual statistics for the cervical screening programme for the year ending 31 March 2020; just as the pandemic hit and services were paused. This data states in 2019/20 the uptake rate was 71.2% with 1,010,963 eligible women being screened in the programme. Younger women and women from the most deprived areas were shown to be less likely to take part in cervical screening, with a 10.5% discrepancy in uptake between the most deprived and least deprived areas in Scotland.^5^ Therefore, even before COVID-19, screening uptake was already low in the most deprived which contributes to an increased risk of developing cervical cancer in this group.

Local data from NHS Ayrshire & Arran for cervical screening samples obtained from 1 November 2019 to 31 October 2020, encompassing the pandemic, show that 11,209 individual tests were carried out, which is 43% of the previous yearly total (25,927 tests).^6^ On average, general practices (GPs) across the area had a reduction of 56% in the number of tests carried out. This suggests that less women have been reached to participate in screening due to COVID-19. It is likely that deprived women will be harder to reach which further widens the disparity between the most and least deprived in their willingness to attend screening and thus exaggerates health inequality. Overall, there has been a significant decrease in the number of women being screened. This is mostly due to services being paused but some women may have been reluctant to attend their rescheduled appointments for various reasons.

## Barriers to screening uptake during COVID-19

The reasons as to why some women may not attend cervical screening are wide ranging and can be challenging to address. These reasons are known as ‘barriers,’ as they impact on a woman’s decision to attend for screening and make her less likely to participate.^7^ The pandemic itself has presented new barriers for women to deal with, but there are some common barriers that existed before COVID-19 that may now be exacerbated. These include embarrassment, fear of the procedure being painful, fear of the possibility of cancer, the opinion that screening is less relevant to certain women, judgement, inconvenience, physical disability, trauma, female genital mutilation (FGM), familiarity with the sample taker and lack of understanding of the procedure.^7^

The barriers to screening during COVID-19 are demonstrated in an online survey by Jo’s Cervical Cancer Trust, which found that 9% of women would not attend cervical screening now due to concern over the virus.^8^ It has been reported that people from black, Asian and minority (BAME) backgrounds may have a higher mortality from COVID-19; therefore, a particularly alarming finding in this survey was that 43% women in this group said they would not attend cervical screening and are more than twice as likely as Caucasian women to delay screening due to safety fears (17.3% of BAME women compared with 8.1%). More promisingly, 43% of all respondents agreed that more information about safety measures in place would make them more likely to engage in cervical screening amid the pandemic. Specific concerns included 29% of women having concern about the safety of visiting a GP surgery, around 36% felt anxiety about their safety as well as the risk of transmission to loved ones, and others mentioned risks of public transport when travelling to an appointment. Some women were uncertain as to whether screening was available; assuming reduced availability and expectation of backlogs. Another worry was staff shortages leading to male healthcare providers carrying out the procedure. It is important to address each of these sources of worry in order to prevent more women missing out on vital screening due to the pandemic.

## Recall and referral to colposcopy

In Scotland, the recall interval and level of intervention needed depends on the presence or absence of HPV as the primary test is high-risk HPV testing. If no HPV is found, the recall interval is now 5 years, regardless of age, which for the 25–49 age group is an extension of 2 years.^1^ If HPV is found, then cytology is performed, and if dyskaryosis is present, then the next stage is colposcopy. As with cervical screening, colposcopy services are subject to prioritization guidance and the BSCCP (British Society for Colposcopy and Cervical Pathology) and the Royal College of Obstetricians & Gynaecologists (RCOG) state that only women who had a recent high-grade moderate cytology, BNC (borderline nuclear change) or possible glandular neoplasia, or suspected invasive cancer should be seen for colposcopy during the pandemic.^9^ Prior to the pandemic, the Government target was for at least 93% of women with these results to be offered colposcopy on a ‘2-week wait’ referral pathway.^10^ However, these referrals may now be triaged within local health boards to prioritize those who need to be seen most urgently. Safety net strategies should also be put in place, such as virtual clinic appointments to minimize attendance to hospital, reassure women and to elicit useful information to aid in triaging.^9^

For women with low-grade or minor cytological abnormalities or persistent HPV infection, the BSCCP and RCOG guidance states that some women can be deferred if the cytological changes are deemed low-risk enough or there is reduced clinic capacity.^9^ Prior to COVID-19, this low-risk group of women were referred on an 18-week pathway and the national target was for at least 99% of these women being offered a colposcopy appointment within 6 weeks of referral.^10^ However, similar to women in the 2-week-wait pathway, these women have been triaged based on their need in line with prioritization guidelines. In summary, some women have experienced postponement of their colposcopy until normal services can resume. The reality of COVID-19 is that when the service works through backlogs of appointments and gets around to seeing certain women, inevitably, cancerous changes may have advanced.

## Improving cervical screening uptake during COVID-19

### Self-sampling

Even before the COVID-19 pandemic, achieving a high percentage uptake of cervical screening was challenging (71.2%) despite efforts being made to break down barriers non-attending women may have.^5^ HPV testing as the primary screening modality, as opposed to cervical cytology, has made self-collection of a sample possible. This would involve the woman obtaining a kit, collecting her own sample with a brush or swab and in the event of abnormal results, the woman is referred for clinical assessment and treatment, similar to bowel screening in Scotland. In 2020, the World Health Organization (WHO) recommended that HPV self-sampling should be made available as an additional option within cervical cancer screening to improve coverage and help reach a global target of 70% coverage of screening by 2030.^11^

A meta-analysis of HPV self-sampling and cervical cancer screening uptake published in the *British Medical Journal* (BMJ) of Global Health overall found women were twice as likely to participate when offered self-sampling compared with standard practices (95% CI: 1.89–2.40).^12^ Another meta-analysis found that 8.7%–39.1% of previous non-attenders ended up participating via self-sampling where this was an option.^13^ As it is known that eligible women who do not attend for screening have an increased risk of cervical cancer, this is an important target population and HPV self-sampling could help reach these women.

In terms of accuracy of HPV self-sampling, a randomized controlled trial in the Netherlands involving over 180,000 women found that 7.4% of samples from the self-collected group tested positive for HPV compared with 7.2% of clinician collected samples and the sensitivity and specificity did not differ between the two methods of collection.^14^ HPV testing is sensitive but not specific because most HPV infections will not lead to cancer. Testing of HPV type is also possible with self-sampling which would address this and would allow risk stratification based on the presence of a high-risk strain.^15^ However, high-risk strains of HPV are common in the population; therefore, a second triage test (currently cytology obtained by a clinician) is required to guide the need for treatment. Self-sampling for cytology would be useful; however, it is not possible as it cannot be ensured that the sample would contain cells from the cervical transformation zone. This is not an issue for HPV self-sampling as a vaginal sample is sufficient to detect HPV but clear instructions and guidance must be provided in the self-sampling kits in order to reduce the risk of women using an incorrect technique and to improve accuracy.

Despite evidence supporting its use, it is important to note that self-sampling methods for HPV testing are not yet approved for U.K.-wide rollout. The pathways to national and global implementation of self-sampling can be challenging: there is concern about how to address loss to follow-up, the design of management algorithms and potential delays in diagnosis. However, it does show promise as a strategy that could reliably prioritize women for further investigation without requiring a face-to-face clinical interaction during COVID-19.

### Education and technology

Even before the pandemic, the need for greater education and public awareness of the importance of cervical screening was ever-present. As the survey by Jo’s Cervical Cancer Trust highlighted, 43% of women made it clear that they would be more likely to attend for screening if more guidance was made available to them. Ensuring women who are due a test are alerted with the use of text messages, phone calls or notices on websites and informing them of what to expect with regards to safety and hygiene measures is key. An important drawback with the use of technology is that those who are most deprived and are less likely to attend screening are also less likely to have access to technology and identifying and reaching this group will be even more challenging due to the pandemic. Particular attention must be given to those who have increased barriers to attending, and methods such as social media, public campaigns, outreach work and literature available in community spaces could all be beneficial.^8^ Although COVID-19 has led to challenges with uptake of cervical screening, the original psychological, physical and cultural barriers to attendance still stand and may now be exacerbated; therefore, reassuring patients and signposting them to support if needed is still as paramount as ever.

An emerging method to engage with patients is telemedicine. For example, women who are deemed high-risk and require treatment at their first visit can have virtual counselling beforehand which ensures the woman’s needs and concerns are understood prior to recommending invasive treatment.^16^ On the contrary, there are some women in the screening population who could safely stop, such as women older than 65 with multiple negative screening results in the past. Virtual consultations and advice around the decision to stop screening could promote access for women who need screening or surveillance; especially important due to the limited cervical screening appointments available currently.

## Conclusion

In conclusion, COVID-19 has had an unprecedented impact on healthcare and the ‘pausing’ of the cervical screening programme has resulted in a backlog of women awaiting screening. It is too soon to know the true figures of how many women experienced a delayed diagnosis of cervical cancer and more studies in future should focus on the long-term effect of this on women’s physical and mental health. COVID-19 has brought the barriers women face to cervical screening to the forefront and although self-sampling shows real promise, in the meantime, we must overcome such barriers through education and counselling. Virtual telemedicine can be used to engage and reassure women in these times of heightened uncertainty. It would be a tragedy if the mortality from COVID-19 was increased due to the delays in cervical cancer screening and diagnosis, but we must hope that this pandemic will lead to opportunities for reflection and new, innovative developments in the delivery of screening services and patient care.
